# Treatment Outcomes and the Safety of Chemoradiotherapy With High-Dose CDDP for Elderly Patients With Head and Neck Squamous Cell Carcinoma: A Propensity Score Matching Study

**DOI:** 10.3389/fsurg.2021.753049

**Published:** 2021-11-23

**Authors:** Jo Omata, Yushi Ueki, Takeshi Takahashi, Ryusuke Shodo, Keisuke Yamazaki, Kohei Saijo, Hisayuki Ota, Takafumi Togashi, Yuichiro Sato, Arata Horii

**Affiliations:** ^1^Department of Otolaryngology Head and Neck Surgery, Niigata University Graduate School of Medical and Dental Sciences, Niigata, Japan; ^2^Department of Head and Neck Surgery, Niigata Cancer Center Hospital, Niigata, Japan

**Keywords:** elderly patients, chemoradiotherapy, propensity score matching, head and neck squamous cell carcinoma, high-dose single-agent cisplatin

## Abstract

**Objective:** We aimed to compare the outcomes and safety of chemoradiotherapy (CRT) between elderly and non-elderly patients with head and neck squamous cell carcinoma (HNSCC). It is difficult to assess the causal effect of age because of possible differences in general conditions among individuals. Therefore, we adjusted the background factors of elderly and non-elderly patients using propensity score matching (PSM).

**Methods:** A total of 146 patients with HNSCC who received CRT were divided into an elderly (≥70 years, *n* = 35) and non-elderly group (<70 years, *n* = 111). Pre-treatment characteristics, including the performance status, Charlson comorbidity index, body mass index, primary site, and TNM stage were adjusted by PSM. We compared the outcomes and safety of CRT with high-dose single-agent cisplatin (CDDP) as well as outcomes following recurrence between the groups, before and after PSM.

**Results:** The total dose of CDDP administered during CRT was significantly lower in the elderly group before PSM. However, it became comparable to the non-elderly group and adverse events did not differ between the groups following PSM, resulting in a comparable CRT completion rate. Overall-, disease specific-, and progression-free survivals of elderly patients were comparable to those of non-elderly patients following PSM. In contrast, elderly patients with recurrence could receive fewer salvage treatments than their non-elderly counterparts, resulting in worse survival.

**Conclusions:** CRT with high-dose CDDP is safe and effective for the treatment of elderly patients with HNSCC. However, salvage treatments can be rarely conducted for elderly patients with a recurrence, considering a deterioration of their general condition.

## Introduction

Head and neck squamous cell carcinoma (HNSCC) is the sixth most common malignancy, with 890,000 new cases and 450,000 deaths per year globally (1, 2). Considering the worldwide increase in life expectancy over the past few decades, the number of elderly patients with HNSCC has increased further. Over 25% of patients with HNSCC are older than 70 years (3, 4). Aging involves the loss of function of multiple organs and systems. Therefore, elderly patients cannot tolerate treatment stress (5). Hence, safety management in the treatment of elderly patients with HNSCC is a clinical issue.

The addition of concurrent high-dose, single-agent cisplatin (CDDP) to radiotherapy (RT) reportedly improves the treatment efficacy (6, 7). Moreover, the National Comprehensive Cancer Network recommends concurrent chemoradiotherapy (CRT) as an organ preservation therapy (8). However, considering the median age of patients in the aforementioned large clinical trials was as low as 56.8–60 years, there is insufficient knowledge about the outcomes and safety of concurrent CRT in elderly patients.

Elderly patients with HNSCC demonstrate greater hematological toxicity, weight loss, lung infection, and fewer chemotherapy courses than non-elderly patients (9–11). The difference in treatment outcomes of concurrent CRT for the elderly patients between reports was possibly because of a patient selection bias (9–12). Moreover, most studies have used multiple chemotherapy regimens rather than a single-agent, high-dose CDDP regimen (9–14). In this multicenter retrospective study, we aimed to compare the safety and treatment outcomes of concurrent CRT using high-dose, single-agent CDDP between elderly and non-elderly patients with HNSCC. To reduce the patient selection bias and to determine the causal effects of age, we performed propensity score matching (PSM), an assessment that adjusts the balance of patient characteristics between two groups (15–18). The outcomes and safety were compared before and after PSM. Moreover, we compared the outcomes following recurrence/metastasis between the groups.

## Materials and Methods

This multicenter retrospective study was approved by the Institutional Review Board of the Niigata University Medical and Dental Hospital (approval number: 2020-0133).

### Patients

**Figure 1:**
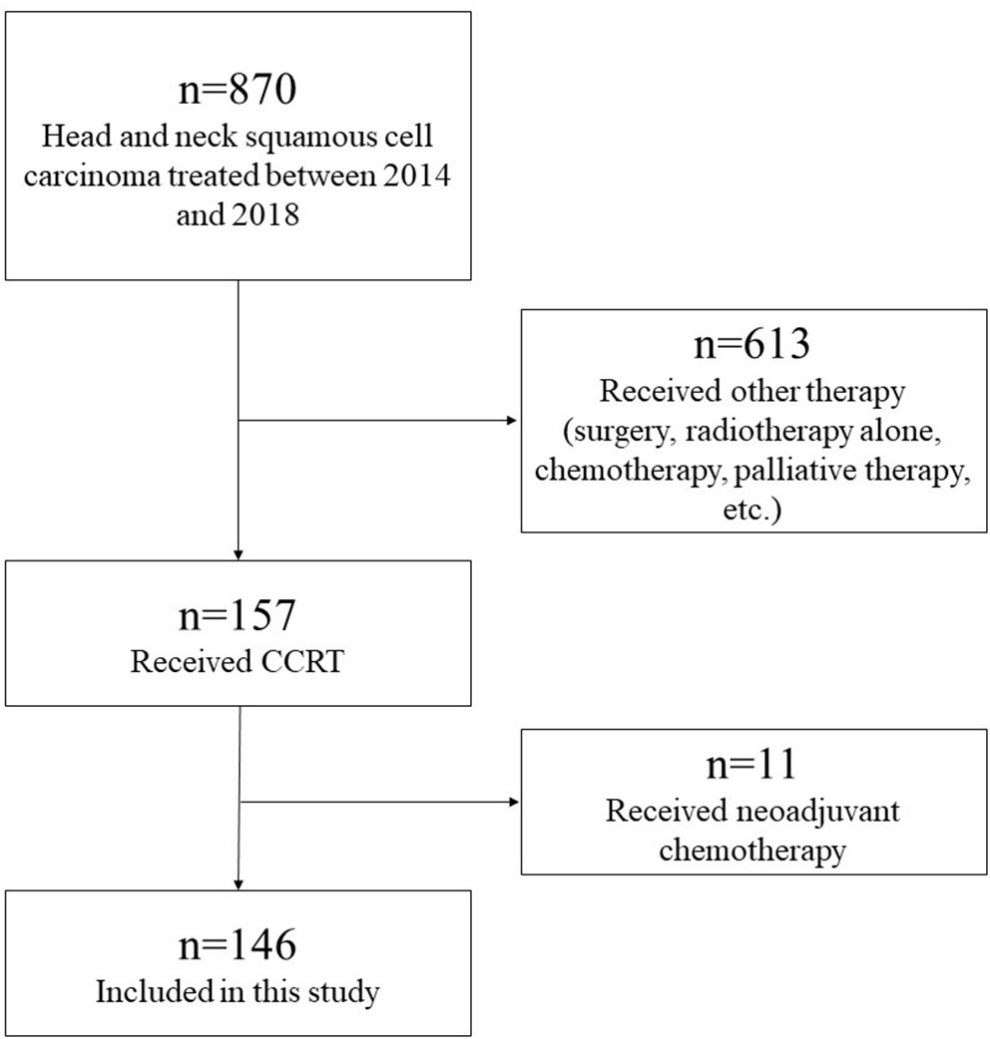
Flow diagram of study participants. A total of 870 HNSCC patients were treated in our institutions. Of the 157 patients treated with concurrent CRT using high-dose CDDP, we excluded 11 patients who received neoadjuvant chemotherapy. A total of 146 patients were included in the study.

A flow diagram of the study participants is shown in Figure 1. The inclusion criteria were as follows: patients with histologically proven HNSCC of the oral cavity, nasopharynx, oropharynx, hypopharynx, and larynx treated with concurrent CRT using high-dose CDDP between January 2014 and December 2018 at the Niigata University Medical and Dental Hospital and Niigata Cancer Center Hospital. A total of 870 HNSCC patients were treated in our institutions. Of the 157 patients treated with concurrent CRT using high-dose CDDP, we excluded 11 patients who received neoadjuvant chemotherapy. A total of 146 patients were included in the study.

We evaluated the following patient characteristics as background factors: age, sex, Eastern Cooperative Oncology Group performance status (PS), comorbid disease, Charlson comorbidity index (CCI),19 body mass index (BMI) at baseline, primary tumor site, and TNM stage according to the Unio Internationalis Contra Cancrum 8th classification. The HPV status in oropharyngeal cancer was evaluated by immunohistochemical staining of the surrogate protein p16; however, it was not evaluated in eight patients.

To assess the effect of aging on safety and treatment outcomes of concurrent CRT, we classified the patients into the following two groups according to their age: elderly (≥70 years, *n* = 35) and non-elderly (<70 years, *n* = 111) (9, 12–14, 19). Furthermore, we compared the rate of recurrence/metastasis following initial concurrent CRT, salvage treatment, and survival after recurrence/metastasis between the groups.

### Treatment Protocols

All patients were hospitalized and received three-dimensional conformal radiation therapy or intensity-modulated radiation therapy on 5 consecutive days per week, at a conventional fractionated daily dose of 2 Gy. The total prescribed radiotherapy dose was 70 Gy. The initial treatment included the primary lesion and whole neck lymph nodes. Following a dose of 40–50 Gy, the treatment was reduced to irradiate the primary lesion and metastatic nodes. All patients were confirmed to have sufficient bone marrow function and no renal dysfunction (estimated glomerular filtration rate ≥60 ml/min) before commencing the treatment. The chemotherapy regimen comprised CDDP (80 mg/m^2^) every 3 weeks, administrated according to the recommend dose for Japanese patients in a report by Matsuyama et al. (20). The CDDP dose was reduced to 60–80% upon observing grade 3–4 adverse events or renal dysfunction. Moreover, the safety of concurrent CRT was assessed using the following markers: the total dose of radiation completed, the total dose of cisplatin received, CRT completion rate, weight loss, the length of hospitalization after treatment, and adverse events according to the Common Terminology Criteria for Adverse Events (CTCAE) Ver. 5.0. We defined the CRT completion rate as the proportion of patients with planned RT completion and three chemotherapy courses during RT delivery, regardless of the CDDP dose. The full-dose CRT completion rate was defined similarly; however, it did not permit dose reduction (20). We assessed the weight loss by the difference in weight at the beginning of CRT and 8 weeks later.

### Follow-Up

We assessed the initial treatment response using imaging tests, such as computed tomography (CT), magnetic resonance imaging (MRI), or positron emission tomography-CT (PET-CT), 8–12 weeks following the end of treatments. As follow-up examinations, CT, MRI, and PET-CT were scheduled every 3–6 months for the first 2–3 years and every 6–12 months thereafter, for a total of 5 years. We evaluated the treatment outcomes by the 2-year overall survival (OS), disease-specific survival (DSS), and progression-free survival (PFS). OS was defined as the time from the initiation of CRT to death from any cause. DSS was defined as the time from the initiation of CRT to death because of disease or toxicity. PFS was defined as the time from the initiation of CRT to either tumor progression or the first evidence for recurrence. In cases of recurrence, the patients underwent salvage treatment depending on their general condition. The treatment outcome for recurrent patients was evaluated using the 1-year OS and DSS.

### Propensity Score Matching

We conducted PSM to avoid confounding differences between the elderly and the non-elderly groups (16–18). The propensity scores were estimated using a logistic regression model based on background factors that might impact the CRT outcomes (18). These factors were as follows: sex, PS, CCI, BMI, primary site, and TNM stage. We performed a one-to-one matched analysis using nearest-neighbor matching on the basis of the estimated propensity scores between the elderly and non-elderly groups. We matched on the logit of the propensity score using calipers of width equal to 0.2 of the standard deviation of the logit of the propensity score (21). A match occurred when a patient in the elderly group had an estimated score within 0.20 of the standard deviation in the non-elderly group. We compared the safety and treatment outcomes of concurrent CRT between the groups before and after PSM.

### Statistical Analyses

We performed Fisher's exact test, independent *t*-test, and Mann-Whitney U test to compare the differences in patient characteristics and treatment safety between the two groups. Survival curves were generated using the Kaplan-Meier method. Moreover, we conducted the log-rank test to compare the differences in OS, DSS, and PFS. The statistical significance was set at *p* < 0.05. All statistical analyses were performed using EZR (Saitama Medical Center, Jichi Medical University, Saitama, Japan), a graphical user interface for R (The R Foundation for Statistical Computing, Vienna, Austria). More precisely, it is a modified version of R commander, designed to add statistical functions frequently used in biostatistics (22).

## Results

### Patient Characteristics

Table 1 summarizes the pre-treatment characteristics of all patients, before and after PSM. Of the 146 patients, 35 (24.0%) were classified to the elderly group. They had worse PS (*p* = 0.011), lower BMI (*p* = 0.014), less p16 positive oropharyngeal cancer (*p* = 0.005), and more hypopharyngeal cancer (*p* = 0.049). Following PSM, the elderly group (*n* = 30) did not reveal statistically significant differences in pre-treatment characteristics from the non-elderly group (*n* = 30) (Table 1).

**Table 1:** Pre-treatment characteristics of all patients before and after propensity score matching.

		**Before propensity score matching**			**After propensity score matching**		
		**(*****n*** **= 146)**			**(*****n*** **= 60)**		
		**Non-elderly**	**Elderly**	***p*-value**	**Non-elderly**	**Elderly**	***p*-value**
		**<70 years**	**≥70 years**		**<70 years**	**≥70 years**	
		**(*n* = 111)**	**(*n* = 35)**		**(*n* = 30)**	**(*n* = 30)**	
Age (median, range), years		63 (37–69)	74 (70–79)	<**0.001**	63.5 (40–69)	74 (70–79)	<**0.001**
Sex	Male	99 (89.2%)	32 (91.4%)	1	30 (100%)	27 (90.0%)	0.237
	Female	12 (10.8%)	3 (8.6%)		0 (0%)	3 (10.0%)	
Performance status	0	100 (90.1%)	25 (71.4%)	**0.011**	24 (80.0%)	24 (80.0%)	1
	1	11 (9.9%)	10 (28.6%)		6 (20.0%)	6 (20.0%)	
Charlson comorbidity index	0, 1	80 (72.1%)	23 (65.7%)	0.525	17 (56.7%)	20 (66.7%)	0.596
	≥2	31 (27.9%)	12 (34.3%)		13 (43.3%)	10 (33.3%)	
BMI (mean ± SD)		22.3 ± 3.1	20.8 ± 3.1	**0.014**	21.1 ± 2.6	21.3 ± 2.9	0.851
Primary tumor site	Oral cavity	1 (0.9%)	0 (0%)	**0.016**	0 (0%)	0 (0%)	0.852
	Nasopharynx	15 (13.5%)	2 (5.7%)		4 (13.3%)	2 (6.7%)	
	Oropharynx (p16+)	26 (23.4%)	1 (2.9%)		1 (3.3%)	1 (3.3%)	
	Oropharynx (p16–)	11 (9.9%)	4 (11.4%)		5 (16.7%)	3 (10.0%)	
	Oropharynx (p16 unknown)	4 (3.6%)	4 (11.4%)		1 (3.3%)	3 (10.0%)	
	Hypopharynx	41 (36.9%)	20 (57.1%)		16 (53.3%)	17 (56.7%)	
	Larynx	13 (11.7%)	4 (11.4%)		3 (10.0%)	4 (13.3%)	
Stage	I	12 (10.8%)	1 (2.9%)	0.48	1 (3.3%)	1 (3.3%)	0.868
	II	24 (21.6%)	8 (22.9%)		8 (26.7%)	6 (20.0%)	
	III	32 (28.8%)	9 (25.7%)		6 (20.0%)	8 (26.7%)	
	IV	43 (38.7%)	17 (48.6%)		15 (50.0%)	15 (50.0%)	

*BMI, body mass index; SD, standard deviation. Bold: statistically significant*.

### Safety and Adverse Events

Table 2 summarizes the safety and adverse events associated with CRT, before and after PSM (CTCAE version 5.0). Before PSM, the elderly group received a significantly lower total dose of CDDP (*p* = 0.045). Moreover, they demonstrated a longer duration of hospitalization following treatment (*p* < 0.001) and more grade three events or higher hyponatremia (*p* = 0.038). Following PSM, there were no significant differences between the groups in the total dose of CDDP, CRT completion rate, and other markers for safety.

**Table 2:** Treatment tolerance and adverse events (CTCAE version 5.0).

		**Before propensity score matching**			**After propensity score matching**		
		**(*****n*** **= 146)**			**(*****n*** **= 60)**		
		**Non-elderly**	**Elderly**	***p*-value**	**Non-elderly**	**Elderly**	***p*-value**
		**<70 years**	**≥70 years**		**<70 years**	**≥70 years**	
		**(*n* = 111)**	**(*n* = 35)**		**(*n* = 30)**	**(*n* = 30)**	
Technique of RT	3DCRT	92 (82.9%)	34 (97.1%)	**0.045**	26 (86.7%)	29 (96.7%)	0.353
	IMRT	19 (17.1%)	1 (2.9%)		4 (13.3%)	1 (3.3%)	
Total dose of RT (mean ± SD), Gy		69.9 ± 0.9	69.7 ± 1.1	0.275	70.0 ± 0.0	69.7 ± 1.2	0.129
Total dose of CDDP (mean ± SD), mg/m^2^		226.0 ± 30.1	212.1 ± 49.0	**0.045**	218.1 ± 39.8	213.9 ± 42.1	0.688
CRT completion rate		90.10%	80.00%	0.14	86.70%	80.00%	0.731
Full-dose CRT completion rate		77.50%	65.70%	0.184	66.70%	63.30%	1
Weight loss (mean ± SD), %		−7.2 ± 5.1	−7.2 ± 4.8	0.985	−6.04 ± 5.3	−7.5 ± 4.3	0.262
Hospitalization after treatment (median, range), days		12 (0–49)	18 (3–63)	<**0.001**	11.5 (1–35)	16.5 (3–63)	0.103
Adverse events (≥Grade 3)	Pharyngeal mucositis	46 (41.4%)	20 (57.1%)	0.121	10 (33.3%)	17 (56.7%)	0.119
	Dermatitis	11 (9.9%)	1 (2.9%)	0.294	1 (3.3%)	1 (3.3%)	1
	Nausea	26 (23.4%)	5 (14.3%)	0.344	4 (13.3%)	5 (16.7%)	1
	Neutropenia	15 (13.5%)	5 (14.3%)	1	3 (10.0%)	5 (16.7%)	0.706
	Hyponatremia	6 (5.4%)	6 (17.1%)	**0.038**	1 (3.3%)	6 (20.0%)	0.103
	Lung infection	5 (4.5%)	2 (5.7%)	0.673	2 (6.7%)	2 (6.7%)	1
Death from treatment toxicity		0 (0%)	0 (0%)	NA	0 (0%)	0 (0%)	NA

*CTCAE, Common Terminology Criteria for Adverse Events; RT, radiotherapy; CRT, chemoradiotherapy; 3DCRT, three-dimensional conformal radiation therapy; IMRT, intensity-modulated radiation therapy; CDDP, cisplatin; SD, standard deviation; NA, not available. Bold: statistically significant*.

### Treatment Outcomes

**Figure 2:**
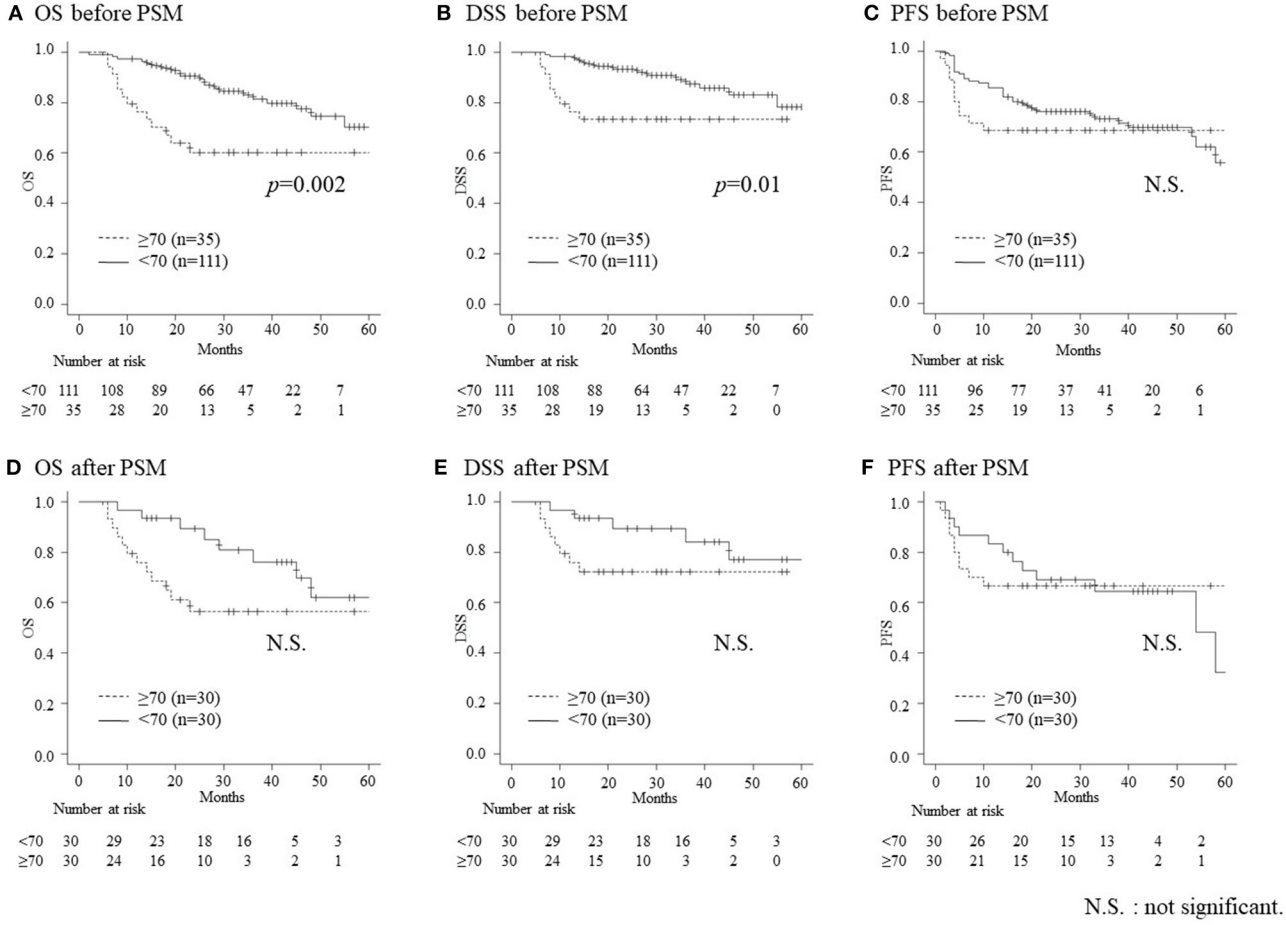
The overall survival (OS), disease-specific survival (DSS), and progression-free survival (PFS) according to the age, before and after propensity score matching (PSM). Before PSM, the OS **(A)** and DSS **(B)** are significantly worse in elderly patients than that in non-elderly patients (60.2% vs. 90.5%, *p* = 0.002 and 73.3% vs. 93.3%, *p* = 0.010, respectively), while there are no significant differences in PFS **(C)** (68.6% vs. 76.0%, *p* = 0.391). Following PSM, there are no significant differences in the OS **(D)**, DSS **(E)**, and PFS **(F)** between the patients (56.4% vs. 89.3%, *p* = 0.052, 72.1% vs. 89.3%, *p* = 0.127 and 66.7% vs. 69.1%, *p* = 0.940, respectively).

The median follow-up time was 31.5 months (range, 2–79 months) and 27.5 months (range, 5–74 months) before and after PSM, respectively. Figure 2 depicts the details of the OS, DSS, and PFS curves using the Kaplan-Meier method. Before PSM, the elderly group had worse 2-year OS and DSS than the non-elderly group (60.2% vs. 90.5%, *p* = 0.002 and 73.3% vs. 93.3%, *p* = 0.010, respectively), while there was no difference in the 2-year PFS between the elderly and non-elderly groups (68.6% vs. 76.0%, *p* = 0.391) (Figures 2A–C). Nine (25.7%) and 14 (12.6%) patients died of primary disease in the elderly and non-elderly group, respectively. In contrast, four (11.4%) and seven (6.3%) patients died of other diseases in the elderly and non-elderly group, respectively. Following PSM, there were no significant differences in 2-year OS (56.4% vs. 89.3%, *p* = 0.052), DSS (72.1% vs. 89.3%, *p* = 0.127), and PFS (66.7% vs. 69.1%, *p* = 0.940) between the elderly and non-elderly groups (Figures 2D–F).

### Recurrence/Metastasis Following Initial Chemoradiotherapy

**Figure 3:**
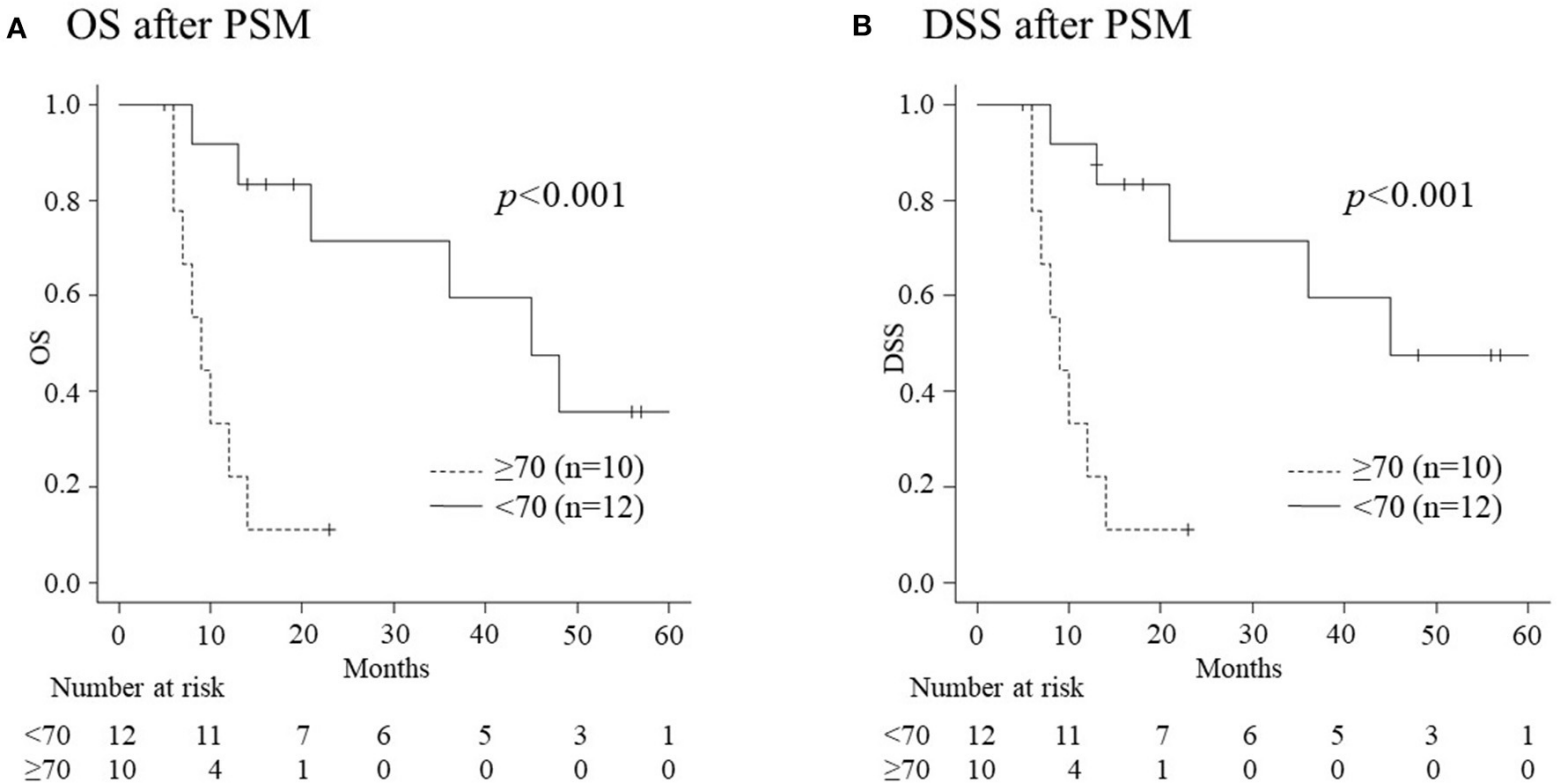
The overall survival (OS) and disease specific survival (DSS) of recurrent patients according to age following propensity score matching (PSM). **(A)** The 1-year OS rate in elderly patients (22.2%) is significantly worse than that in non-elderly patients (91.7%) (*p* < 0.001). **(B)** The 1-year rate of DSS in elderly patients (22.2%) is significantly worse than that in non-elderly patients (91.7%) (*p* < 0.001).

Of the 146 patients who received CRT for advanced HNSCC, 44 developed recurrence/metastasis following the initial CRT: 11 elderly and 33 non-elderly patients. There were no differences in the occurrence of recurrence/metastasis between the groups (31.4% vs. 29.7%, *p* = 0.836). Moreover, there were no significant differences between the groups in relation to the pre-treatment characteristics of patients who developed recurrence/metastasis, both before and after PSM (Table 3). Table 4 summarizes the treatment safety and adverse events associated with CRT in patients with recurrence/metastasis. The elderly group experienced longer hospitalization before PSM (*p* = 0.018). However, there were no significant differences in the CRT completion rate, the length of hospitalization after treatment, adverse events, and other treatment safety between the two groups following PSM. Table 5 presents the recurrent site and salvage treatment administered to patients with recurrence. More patients in the elderly group were unable to receive salvage treatment than those in the non-elderly group, both before (*p* < 0.001) and after PSM (*p* = 0.02). This can be attributed to poor general conditions. All patients who could not receive salvage treatment in the elderly group revealed tumor progression or recurrence within 5 months following the initiation of CRT. They were considered unsuitable for salvage treatments because of their inability for oral intake and poor PS. Figure 3 depicts the details of the OS and DSS curves using the Kaplan-Meier method for patients with recurrence/metastasis following PSM. Elderly patients with recurrence/metastasis had worse 1-year OS and DSS than their non-elderly counterparts (22.2% vs. 91.7%, *p* < 0.001 and 22.2% vs. 91.7%, *p* < 0.001, respectively).

**Table 3:** Pre-treatment characteristics of patients who developed recurrence/metastasis.

		**Before propensity score matching**			**After propensity score matching**		
		**(*****n*** **= 44)**			**(*****n*** **= 22)**		
		**Non-elderly**	**Elderly**	***p*-value**	**Non-elderly**	**Elderly**	***p*-value**
		**<70 years**	**≥70 years**		**<70 years**	**≥70 years**	
		**(*n* = 33)**	**(*n* = 11)**		**(*n* = 12)**	**(*n* = 10)**	
Age (median, range), years		63.0 (37–69)	74.0 (70–77)	**<0.001**	64.5 (51–69)	74.5 (70–77)	<**0.001**
Sex	Male	29 (87.9%)	10 (90.9%)	1	12 (100%)	9 (90.0%)	0.455
	Female	4 (12.1%)	1 (9.1%)		0 (0%)	1 (10.0%)	
Performance status	0	25 (75.8%)	7 (63.6%)	0.457	8 (66.7%)	7 (70.0%)	1
	1	8 (24.2%)	4 (36.4%)		4 (33.3%)	3 (30.0%)	
Charlson comorbidity index	0, 1	24 (72.7%)	9 (81.8%)	0.701	6 (50.0%)	8 (80.0%)	0.204
	≥2	9 (27.3%)	2 (18.2%)		6 (50.0%)	2 (20.0%)	
BMI (mean ± SD)		22.2 ± 3.0	20.6 ± 3.2	0.157	21.3 ± 2.9	21.0 ± 3.1	0.826
Primary tumor site	Oral cavity	1 (3.0%)	0 (0%)	0.308	0 (0%)	0 (0%)	0.339
	Nasopharynx	4 (12.1%)	0 (0%)		1 (8.3%)	0 (0%)	
	Oropharynx (p16+)	6 (18.2%)	0 (0%)		0 (0%)	0 (0%)	
	Oropharynx (p16–)	6 (18.2%)	2 (18.2%)		4 (33.3%)	1 (10.0%)	
	Oropharynx (p16 unknown)	0 (0%)	1 (9.1%)		0 (0%)	1 (10.0%)	
	Hypopharynx	14 (42.4%)	7 (63.6%)		7 (58.3%)	7 (70.0%)	
	Larynx	2 (6.1%)	1 (9.1%)		0 (0%)	1 (10.0%)	
Stage	I	1 (3.0%)	0 (0%)	0.912	0 (0%)	0 (0%)	1
	II	6 (18.2%)	1 (9.1%)		2 (16.7%)	1 (10.0%)	
	III	7 (21.2%)	2 (18.2%)		1 (8.3%)	1 (10.0%)	
	IV	19 (57.6%)	8 (72.7%)		9 (75.0%)	8 (80.0%)	

*BMI, body mass index; SD, standard deviation. Bold: statistically significant*.

**Table 4:** Treatment tolerance and adverse events of patients had recurrence.

		**Before propensity score matching**			**After propensity score matching**		
		**(*****n*** **= 44)**			**(*****n*** **= 22)**		
		**Non-elderly**	**Elderly**	***p*-value**	**Non-elderly**	**Elderly**	***p*-value**
		**<70 years**	**≥70 years**		**<70 years**	**≥70 years**	
		**(*n* = 33)**	**(*n* = 11)**		**(*n* = 12)**	**(*n* = 10)**	
Technique of RT	3DCRT	29 (87.9%)	11 (100%)	0.558	11 (91.7%)	10 (100%)	1
	IMRT	4 (12.1%)	0 (0%)		1 (8.3%)	0 (0%)	
Total dose of RT (mean ± SD), Gy		69.7 ± 1.6	69.1 ± 1.9	0.296	70.0 ± 0.0	69.0 ± 1.9	0.088
Total dose of CDDP (mean ± SD), mg/m^2^		219.6 ± 39.9	194.9 ± 44.6	0.091	196.0 ± 54.6	190.4 ± 44.3	0.797
CRT completion rate		84.80%	54.50%	0.09	66.70%	50.00%	0.666
Full-dose CRT completion rate		72.70%	45.50%	0.144	50.00%	40.00%	0.691
Weight loss (mean ± SD), %		−6.4 ± 5.2	−5.1 ± 5.0	0.476	−5.11 ± 5.7	−5.12 ± 5.3	0.998
Hospitalization after treatment (median, range), days		14 (4–37)	35 (3–63)	**0.018**	9.0 (5–35)	36.5 (3–63)	0.064
Adverse events (≥Grade 3)	Pharyngeal mucositis	12 (36.4%)	6 (54.5%)	0.314	5 (41.7%)	5 (50.0%)	1
	Dermatitis	5 (15.2%)	1 (9.1%)	1	0 (0%)	1 (10.0%)	0.455
	Nausea	7 (21.2%)	2 (18.2%)	1	1 (8.3%)	2 (20.0%)	0.571
	Neutropenia	4 (12.1%)	2 (18.2%)	0.63	1 (8.3%)	2 (20.0%)	0.571
	Hyponatremia	2 (6.1%)	3 (27.3%)	0.091	0 (3.0%)	3 (30.0%)	0.078
	Lung infection	2 (6.1%)	1 (9.1%)	1	1 (8.3%)	1 (10.0%)	1
Death from treatment toxicity		0 (0%)	0 (0%)	NA	0 (0%)	0 (0%)	NA

*RT, radiotherapy; CRT, chemoradiotherapy; 3DCRT, three-dimensional conformal radiation therapy; IMRT, intensity-modulated radiation therapy; CDDP, cisplatin; SD, standard deviation; NA, not available. Bold: statistically significant*.

**Table 5:** Recurrent site and salvage treatment of patients with recurrence.

		**Before propensity score matching**			**After propensity score matching**		
		**(*****n*** **= 44)**			**(*****n*** **= 22)**		
		**Non-elderly**	**Elderly**	***p*-value**	**Non-elderly**	**Elderly**	***p*-value**
		**<70 years**	**≥70 years**		**<70 years**	**≥70 years**	
		**(*n* = 33)**	**(*n* = 11)**		**(*n* = 12)**	**(*n* = 10)**	
Recurrent site	Locoregional recurrence	17 (51.5%)	11 (100%)	**0.003**	8 (66.7%)	10 (100%)	0.096
	Distant metastasis only	16 (48.5%)	0 (0%)		4 (33.3%)	0 (0%)	
Salvage treatment	Surgery	5 (15.2%)	1 (9.1%)	1	2 (16.7%)	1 (10.0%)	1
	RT to metastatic site	5 (15.2%)	1 (9.1%)	1	3 (25.0%)	1 (10.0%)	0.594
	Chemotherapy	19 (57.6%)	2 (18.2%)	**0.036**	5 (41.7%)	2 (20.0%)	0.381
	No treatment due to poor general condition	2 (6.1%)	6 (54.5%)	**0.001**	1 (8.3%)	6 (60%)	**0.02**
	No treatment due to refusal	2 (6.1%)	1 (9.1%)	1	1 (8.3%)	0 (0%)	1

*RT, radiotherapy. Bold: statistically significant*.

## Discussion

This is the first report on the safety and outcomes of concurrent CRT with high-dose CDDP for the treatment of elderly patients with HNSCC and non-elderly controls using PSM. Following PSM, which controlled for sex, PS, CCI, BMI, primary site, and TNM stage, background factors in the elderly group, including a total dose of CDDP, the duration of hospitalization following treatment, and hyponatremia became non-significant compared with those in the non-elderly group (Table 2). The CRT completion rate and treatment outcomes (OS, DSS, and PFS) (Figure 2) in elderly patients were comparable to those in non-elderly patients following PSM. In contrast, fewer elderly patients could receive salvage treatments for recurrent and metastatic diseases following initial CRT, despite PSM. This can be attributed to poor general conditions, which resulted in poorer treatment outcomes for recurrent diseases among elderly patients than their non-elderly counterparts (Figure 3).

We conducted PSM to adjust for background factors, including sex, PS, CCI, BMI, primary site, and TNM stage between the groups. PSM is now a widely accepted statistical approach that enables robust comparisons (15). Moreover, several studies have focused on the effects of aging using PSM to avoid confounding differences because of a variability in background factors between elderly and younger patients (16–18). There were statistically significant differences in the PS, BMI, and primary tumor site, including p16 positive oropharynx cancer before PSM, all of which were corrected following PSM (Table 1). PS and p16 positive oropharyngeal cancer were considered to influence the prognosis of patients with HNSCC (23, 24). An adjustment of the aforementioned factors helped us analyze the significance of aging on the treatment safety and outcomes of concurrent CRT. Following PSM, the total dose of CDDP, the duration of hospitalization following treatment, and hyponatremia, all of which demonstrated significant differences between the elderly and non-elderly patients, became non-significant.

Michal et al. reported that elderly patients could receive less cycles of chemotherapy and demonstrated greater incidence of neutropenia and unplanned hospitalization than younger patients who underwent CRT for locally advanced HNSCC (9). In contrast, Nguyen et al. reported that there were no significant differences in the treatment completion rate of CRT for locally advanced HNSCC between elderly and younger patients (12). These discrepant conclusions could be attributed to differences in background factors other than age, which in turn were controlled by PSM in the present study. Moreover, previous reports used a multi-agent chemotherapy regimen, which could also affect the safety of CRT. To address the aforementioned problems, we used a single regimen of high-dose CDDP (80 mg/m^2^) together with RT. For a high–dose CDDP regimen, 100 mg/m^2^ CDDP is considered as the standard dose for advanced HNSCC; however, the CRT completion rate with the 100 mg/m^2^ regimen was reportedly lower for Japanese patients than for those in Western countries (20). Matsuyama et al. demonstrated that CRT using triweekly 80 mg/m^2^ CDDP achieved favorable CRT completion rate and non-inferiority when compared with CRT using 100 mg/m^2^ in our previous phase I/II studies, suggesting that 80 mg/m^2^ CDDP is the standard for high-dose CDDP regimen in Japanese patients (20). As shown in Table 2, there were no significant differences in the treatment safety and CRT completion rate between the elderly and the non-elderly groups. Furthermore, there were no treatment-related deaths in the elderly group (Table 2). Our findings suggest that CRT using high-dose CDDP might be safe and well-tolerated in elderly patients with HNSCC and good general conditions.

In the present study, there were no significant differences in the 2-year OS, DSS, and PFS between the groups following PSM, despite worse OS and DSS in the elderly group before PSM (Figure 2). This result was because of an adjustment of background factors, such as sex, PS, CCI, BMI, primary site, and TNM stage by PSM. Controlling the above-mentioned factors by PSM resulted in an adjustment of differences in the total dose of administrable CDDP and adverse events, such as hyponatremia, which might have affected the treatment outcomes (Table 2).

In a meta-analysis of chemotherapy in head and neck cancer (MACH-NC), the effect of concomitant chemoradiotherapy was decreased with increasing age (19). While the MACH-NC included the largest prospective cohort analyzing CRT, elderly patients only comprised 7% of all patients. Furthermore, it included patients treated in the late 1960s. Treatment strategies and supportive care have advanced since the 1960s. Therefore, the application of the MACH-NC results to the present day may be inappropriate. Michal et al. (9) and Nguyen et al. (12) reported on comparable treatment outcomes of CRT between patients with HNSCC older than 70 years and younger controls in the past decade. Amini et al. (13) and Ward et al. (14) reported on improved OS with CRT, compared to RT only, in patients older than 70 years, based on the US National Cancer Database. In the present study, there were no significant differences in the treatment outcomes between elderly and non-elderly patients following PSM (Figure 1). Our findings suggest that elderly patients should not be considered unsuitable for CRT because of age alone and that CRT using high-dose CDDP may be one of a good treatment option for elderly patients with HNSCC with sufficient general conditions.

Eleven of the 35 elderly patients (31.4%) and 33 of the 111 (29.7%) non-elderly patients developed recurrence/metastasis, revealing no differences between the groups. Despite PSM, greater number of patients were unable to receive any type of salvage treatment for recurrence/metastasis because of poor general condition in the elderly group than those in the non-elderly group (60% vs. 8.3%, *p* = 0.02) (Table 5). Consequently, the 1-year OS and DSS of patients who developed recurrence/metastasis were significantly worse in the elderly group than in the non-elderly group, not only before but also after PSM (Figure 3). All elderly patients who were unable to receive salvage treatment reported early recurrence following CRT, and their general condition had not completely recovered. The aforementioned data suggest that the prognosis of elderly patients with recurrence/metastasis is poor due to the difficulty in implementing salvage treatments. No significant background factors could predict recurrence or metastasis (Table 3). This necessitates a progress of intensive supportive care to minimize the deterioration of general conditions by CRT (25–27) and less invasive salvage treatments, such as immune checkpoint inhibitors (28–31) to improve the prognosis of elderly patients with recurrence/metastasis.

Several limitations of this study should also be acknowledged. First, the CDDP dose of 80 mg/m^2^ in this study is different from the CDDP dose of 100 mg/m^2^ adopted in many Western institutes due to survival benefits in the Japanese population. Therefore, it may be difficult to generalize the results of this study for all elderly HNSCC patients, including those in Western countries. Second, a small sample size from two institutes is an obvious limitation. Especially, it was difficult to accumulate elderly patients with sufficient general conditions tolerable for concurrent CRT. While PSM minimized the selection bias, it also reduced the sample size. To resolve these issues, further prospective studies with a large number of patients are warranted.

## Conclusion

After controlling for background factors using PSM, the safety and outcomes of concurrent CRT with high-dose CDDP for elderly patients with HNSCC were comparable to those for non-elderly patients, suggesting that healthy elderly patients should be treated with the aforementioned technique. However, salvage treatments could not be often conducted for recurrence/metastasis in the elderly group because of a deterioration of their general conditions.

## Data Availability Statement

The original contributions presented in the study are included in the article/supplementary material, further inquiries can be directed to the corresponding author.

## Ethics Statement

This multicenter retrospective study was approved by the Institutional Review Board of the Niigata University Medical and Dental Hospital (approval number: 2020-0133). The patients/participants provided their written informed consent to participate in this study.

## Author Contributions

JO, YU, and AH: conception and design and drafting of the manuscript. JO, YU, TTa, HO, RS, KY, KS, TTo, and YS: analysis and interpretation of data. YU: had full access to all the data in the study and takes responsibility for the integrity of the data and the accuracy of the data analysis. All authors: final approval of the version to be published.

## Conflict of Interest

The authors declare that the research was conducted in the absence of any commercial or financial relationships that could be construed as a potential conflict of interest.

## Publisher's Note

All claims expressed in this article are solely those of the authors and do not necessarily represent those of their affiliated organizations, or those of the publisher, the editors and the reviewers. Any product that may be evaluated in this article, or claim that may be made by its manufacturer, is not guaranteed or endorsed by the publisher.

## References

[1] Bray F Ferlay J Soerjomataram I Siegel RL Torre LA Jemal A. Global cancer statistics 2018: GLOBOCAN estimates of incidence and mortality worldwide for 36 cancers in 185 countries. CA Cancer J Clin. (2018) 68:394–424. 10.3322/caac.21492 30207593

[2] Johnson DE Burtness B Leemans CR Lui VWY Bauman JE Grandis JR. Head and neck squamous cell carcinoma. Nat Rev Dis Primers. (2020) 6:1–22. 10.1038/s41572-020-00224-3 33243986 PMC7944998

[3] Neve M Jameson MB Govender S Hartopeanu C. Impact of geriatric assessment on the management of older adults with head and neck cancer: a pilot study. J Geriatr Oncol. (2016) 7:457–62. 10.1016/j.jgo.2016.05.006 27313080

[4] Grénman R Chevalier D Gregoire V Myers E Rogers S. Treatment of head and neck cancer in the elderly: European Consensus (panel 6) at the EUFOS Congress in Vienna 2007. Eur Arch Otorhinolaryngol. (2010) 267:1619–21. 10.1007/s00405-010-1263-6 20454970

[5] Balducci L. Aging, frailty, and chemotherapy. Cancer Control. (2007) 14:7–12. 10.1177/107327480701400102 17242666

[6] Adelstein DJ Li Y Adams GL Wagner H Jr Kish JA Ensley JF. An intergroup phase III comparison of standard radiation therapy and two schedules of concurrent chemoradiotherapy in patients with unresectable squamous cell head and neck cancer. J Clin Oncol. (2003) 21:92–8. 10.1200/JCO.2003.01.008 12506176

[7] Forastiere AA Goepfert H Maor M Pajak TF Weber R Morrison W. Concurrent chemotherapy and radiotherapy for organ preservation in advanced laryngeal cancer. N Engl J Med. (2003) 349:2091–8. 10.1056/NEJMoa031317 14645636

[8] National Comprehensive Cancer Network. Head and Neck Cancers (version 2.2021). Available online at: https://www.nccn.org/professionals/physician_gls/pdf/head-and-neck.pdf (accessed April 16, 2021).

[9] Michal SA Adelstein DJ Rybicki LA Rodriguez CP Saxton JP Wood BG. Multi-agent concurrent chemoradiotherapy for locally advanced head and neck squamous cell cancer in the elderly. Head Neck. (2012) 34:1147–52. 10.1002/hed.21891 22021098

[10] Chang PH Yeh KY Huang JS Chen EY Yang SW Wang CH. Chemoradiotherapy in elderly patients with advanced head and neck cancer under intensive nutritional support. Asia Pac J Clin Oncol. (2015) 11:228–35. 10.1111/ajco.12323 25535674

[11] Merlano MC Monteverde M Colantonio I Denaro N Lo Nigro C Natoli G. Impact of age on acute toxicity induced by bio- or chemo-radiotherapy in patients with head and neck cancer. Oral Oncol. (2012) 48:1051–7. 10.1016/j.oraloncology.2012.05.001 22658677

[12] Nguyen NP Vock J Chi A Vinh-Hung V Dutta S Ewell L. Impact of intensity-modulated and image-guided radiotherapy on elderly patients undergoing chemoradiation for locally advanced head and neck cancer. Strahlenther Onkol. (2012) 188:677–83. 10.1007/s00066-012-0125-0 22659942

[13] Amini A Jones BL McDermott JD Serracino HS Jimeno A Raben D. Survival outcomes with concurrent chemoradiation for elderly patients with locally advanced head and neck cancer according to the National Cancer Data Base. Cancer. (2016) 122:1533–43. 10.1002/cncr.29956 26969811

[14] Ward MC Reddy CA Adelstein DJ Koyfman SA. Use of systemic therapy with definitive radiotherapy for elderly patients with head and neck cancer: a National Cancer Data Base analysis. Cancer. (2016) 122:3472–83. 10.1002/cncr.30214 27504955

[15] Rosenbaum PR Rubin DB. The central role of the propensity score in observational studies for causal effects. Biometrika. (1983) 70:41–55. 10.1093/biomet/70.1.41

[16] Takagi K Umeda Y Yoshida R Nobuoka D Kuise T Fushimi T. The outcome of complex hepato-pancreato-biliary surgery for elderly patients: a propensity score matching analysis. Dig Surg. (2019) 36:323–30. 10.1159/000489826 29945139 PMC6604258

[17] Sui X Zhao H Wang J Yang F Yang F Li Y. Outcome of VATS lobectomy for elderly non-small cell lung cancer: a propensity score-matched study. Ann Thorac Cardiovasc Surg. (2015) 21:529–35. 10.5761/atcs.oa.15-00126 26439136 PMC4905030

[18] Kanda M Koike M Tanaka C Kobayashi D Hayashi M Yamada S. Feasibility of subtotal esophagectomy with systematic lymphadenectomy in selected elderly patients with esophageal cancer; a propensity score matching analysis. BMC Surg. (2019) 19:1–8. 10.1186/s12893-019-0617-2 31615499 PMC6792188

[19] Pignon JP le Maître A Maillard E Bourhis J. Meta-analysis of chemotherapy in head and neck cancer (MACH-NC): an update on 93 randomised trials and 17,346 patients. Radiother Oncol. (2009) 92:4–14. 10.1016/j.radonc.2009.04.014 19446902

[20] Matsuyama H Yamazaki K Okabe R Ueki Y Shodo R Omata J. Multicenter phase I/II study of chemoradiotherapy with high-dose CDDP for head and neck squamous cell carcinoma in Japan. Auris Nasus Larynx. (2018) 45:1086–92. 10.1016/j.anl.2018.02.008 29567334

[21] Austin PC. Optimal caliper widths for propensity-score matching when estimating differences in means and differences in proportions in observational studies. Pharm Stat. (2011) 10:150–61. 10.1002/pst.433 20925139 PMC3120982

[22] Kanda Y. Investigation of the freely available easy-to-use software ‘EZR' for medical statistics. Bone Marrow Transplant. (2013) 48:452–8. 10.1038/bmt.2012.244 23208313 PMC3590441

[23] Wang JR Habbous S Espin-Garcia O Chen D Huang SH Simpson C. Comorbidity and performance status as independent prognostic factors in patients with head and neck squamous cell carcinoma. Head Neck. (2016) 38:736–42. 10.1002/hed.23947 25521753

[24] Ang KK Harris J Wheeler R Weber R Rosenthal DI Nguyen-Tân PF. Human papillomavirus and survival of patients with oropharyngeal cancer. N Engl J Med. (2010) 363:24–35. 10.1056/NEJMoa0912217 20530316 PMC2943767

[25] Isenring EA Capra S Bauer JD. Nutrition intervention is beneficial in oncology outpatients receiving radiotherapy to the gastrointestinal or head and neck area. Br J Cancer. (2004) 91:447–52. 10.1038/sj.bjc.6601962 15226773 PMC2409852

[26] Carnaby-Mann G Crary MA Schmalfuss I Amdur R. “Pharyngocise”: randomized controlled trial of preventative exercises to maintain muscle structure and swallowing function during head-and-neck chemoradiotherapy. Int J Radiat Oncol Biol Phys. (2012) 83:210–9. 10.1016/j.ijrobp.2011.06.1954 22014959

[27] Samuel SR Maiya AG Fernandes DJ Guddattu V Saxena PUP Kurian JR. Effectiveness of exercise-based rehabilitation on functional capacity and quality of life in head and neck cancer patients receiving chemo-radiotherapy. Support Care Cancer. (2019) 27:3913–20. 10.1007/s00520-019-04750-z 30919154 PMC6728220

[28] Ferris RL Blumenschein G Jr Fayette J Guigay J Colevas AD Licitra L. Nivolumab for recurrent squamous-cell carcinoma of the head and neck. N Engl J Med. (2016) 375:1856–67. 10.1056/NEJMoa1602252 27718784 PMC5564292

[29] Ferris RL Blumenschein G Jr Fayette J Guigay J Colevas AD Licitra L. Nivolumab vs investigator's choice in recurrent or metastatic squamous cell carcinoma of the head and neck: 2-year long-term survival update of CheckMate 141 with analyses by tumor PD-L1 expression. Oral Oncol. (2018) 81:45–51. 10.1016/j.oraloncology.2018.04.008 29884413 PMC6563923

[30] Burtness B Harrington KJ Greil R Soulières D Tahara M de Castro G Jr. Pembrolizumab alone or with chemotherapy versus cetuximab with chemotherapy for recurrent or metastatic squamous cell carcinoma of the head and neck (KEYNOTE-048): a randomised, open-label, phase 3 study. Lancet. (2019) 394:1915–28. 10.1016/S0140-6736(19)32591-7 31679945

[31] Saba NF Blumenschein G Jr Guigay J Licitra L Fayette J Harrington KJ. Nivolumab versus investigator's choice in patients with recurrent or metastatic squamous cell carcinoma of the head and neck: efficacy and safety in CheckMate 141 by age. Oral Oncol. (2019) 96:7–14. 10.1016/j.oraloncology.2019.06.017 31422216 PMC7723820

